# Supplementary material for: Solubility Determination of c-Met Inhibitor in Solvent Mixtures and Mathematical Modeling to Develop Nanosuspension Formulation
Source: Molecules. 2021 Jan 13;26(2):390. doi: 10.3390/molecules26020390 (PMC7828412; doi:10.3390/molecules26020390)
Supplement: Supplementary file 1 [file molecules-26-00390-s001.pdf]

molecules
MDPI

Solubility determination of c-Met inhibitor in solvent mixtures and mathematical modeling to develop nanosuspension formulation

Maharjan Ravi[1], Tripathi Julu[1], Nam Ah Kim[1,2], Kyeung Eui Park[2], Seong Hoon Jeong[1*]

[1]BK21 FOUR Team and Integrated Research Institute for Drug Development,

College of Pharmacy, Dongguk University, Gyeonggi 10326, Republic of Korea

[2]R&D Center, ABION Inc., Seoul 08394, Republic of Korea

Authors' addresses

Maharjan Ravi, MS, email: raavii@dgu.ac.kr

Tripathi Julu, MS, email: julutripathi@gmail.com

Kyeung Eui Park, PhD, email: pku1218@abionbio.com

* To whom correspondence should be addressed.

Nam Ah Kim, PhD, email: namah87@dongguk.edu

Seong Hoon Jeong, PhD, email: shjeong@dongguk.edu

Tel: (82) 31-961-5225

Submitted to *Molecules*

Table S1. Experimental mole fraction solubility of drug in organic solvents over a temperature range of 298.15-318.15 K

Solvent kept at temperature (K)	X_{exp}*10^{-4}	X_{cal}*10^{-4} (Modified AM)	X_{cal}*10^{-4} (VHM)	X_{cal}*10^{-4} (BKM)
		Water		
298.15	0.028	0.028	0.028	0.028
303.15	0.033	0.033	0.033	0.033
308.15	0.039	0.038	0.039	0.039
313.15	0.044	0.045	0.045	0.046
318.15	0.054	0.053	0.053	0.053
		Methanol		
298.15	0.386	0.375	0.398	0.397
303.15	0.533	0.499	0.515	0.514
308.15	0.673	0.646	0.660	0.659
313.15	0.826	0.814	0.840	0.840
318.15	1.055	1.000	1.061	1.066
		Ethanol		
298.15	2.171	2.114	2.172	2.166
303.15	2.916	3.074	3.015	3.015
308.15	4.286	4.281	4.140	4.155
313.15	6.058	5.726	5.629	5.670
318.15	7.173	7.373	7.580	7.670
		1-Propanol		
298.15	7.114	7.115	7.193	7.199
303.15	8.239	8.183	8.139	8.112
308.15	9.083	9.267	9.172	9.151
313.15	10.555	10.344	10.296	10.308
318.15	11.312	11.388	11.517	11.609
		2-Propanol		
298.15	2.580	2.553	2.572	2.570
303.15	3.245	3.317	3.303	3.296
308.15	4.220	4.239	4.206	4.198
313.15	5.483	5.335	5.315	5.313
318.15	6.524	6.616	6.667	6.685
		1-Butanol		
298.15	8.278	8.455	8.269	8.307
303.15	9.046	9.152	8.984	8.980
308.15	9.549	9.906	9.734	8.717
313.15	10.660	10.721	10.521	10.053
318.15	11.322	11.602	11.343	11.427
		2-Butanol		
298.15	4.083	3.984	4.213	4.200
303.15	5.652	5.520	5.554	5.541
308.15	7.434	7.320	7.255	7.247
313.15	9.640	9.316	9.398	9.408
318.15	11.632	11.407	12.074	12.131
		Acetonitrile		
298.15	6.086	5.969	6.028	6.011
303.15	6.312	6.504	6.520	6.490
308.15	7.204	7.032	7.035	7.013

313.15	7.672	7.549	7.571	7.590
318.15	8.020	8.048	8.130	8.226
		Acetone		
298.15	9.798	9.658	9.533	9.523
303.15	10.141	10.528	10.591	10.559
308.15	11.788	11.589	11.726	11.697
313.15	13.050	12.876	12.941	12.964
318.15	14.269	14.428	14.237	14.373
		Ethyl acetate		
298.15	6.437	6.450	6.443	4.569
303.15	6.777	6.813	6.781	4.960
308.15	7.161	7.192	7.149	5.274
313.15	7.589	7.587	7.555	5.680
318.15	7.958	7.998	8.001	6.127
		THP		
298.15	20.584	20.575	20.706	20.657
303.15	24.069	24.976	24.462	24.438
308.15	29.311	29.590	28.744	28.667
313.15	34.665	34.264	33.601	33.620
318.15	37.906	38.832	39.086	39.351

Table S2. Application of Fedors′ method to estimate internal energy, molar volume, and Hildebrand solubility parameter of ABN401.

Group	Group number	Δ_{ei} ($kJ\ mol^{-1}$)	Δ_{vi} ($cm^3\ mol^{-1}$)
CH_3	2	2 × 4.71 = 9.42	2 × 33.5 = 67
CH_2	8	8 × 4.94 = 39.52	8 × 16.1 = 128.8
–CH=	9	9 × 3.43 = 30.87	9 × 13.5 = 121.5
CH	1	3.43	–1
C=	8	8 × 4.30 = 34.40	8 × –5.5 = –44
N	5	5 × 4.18 = 20.9	5 × –9 = –45
–N=	7	7 × 11.71 = 81.97	7 × 5 = 35
O	1	3.35	3.8
Phenylene (p)	1	31.92	52.4
Conjugated double bonds	9	9 × 1.67 = 15.03	9 × –2.2 = –19.8
Ring closure 5 or more atoms	6	6 × 1.05 = 6.30	6 × 16 = 96
Total		277.11	394.7
Solubility parameter		$(277110/394.7)^{1/2} = 26.5\ MPa^{1/2}$	

Table S3. Solvatochromic parameters (α, β, and π*) and Hildebrand solubility parameter (δ_H) for solvents.

Solvents	α	β	π*	δ_H (MPa$^{1/2}$)
Water	1.17	0.47	1.09	47.82
Methanol	0.98	0.66	0.60	29.61
Ethanol	0.86	0.75	0.54	26.13
1-Propanol	0.84	0.90	0.52	24.45
2-Propanol	0.76	0.84	0.48	23.58
1-Butanol	0.84	0.84	0.47	23.35
2-Butanol	0.69	0.80	0.40	28.70
Acetonitrile	0.19	0.40	0.75	24.40
Acetone	0.08	0.43	0.71	19.95
Ethyl acetate	0.00	0.45	0.55	18.20
THP	0.00	–	–	22.30

*Data obtained from the references [S1, S2, and S3]

Table S4. Experimental mole fraction solubility (X_{exp}*10^{-4}) values of ABN401 in THP mixture at different temperatures.

x_2	298.15 K	303.15 K	308.15 K	313.15 K	318.15 K
0	0.028	0.033	0.039	0.044	0.054
0.1	0.105	0.117	0.134	0.146	0.168
0.2	0.161	0.199	0.243	0.281	0.294
0.3	0.279	0.392	0.470	0.561	0.689
0.4	0.796	0.812	0.948	1.341	1.925
0.5	1.943	2.746	3.346	4.123	4.416
0.6	4.691	5.366	6.205	7.103	7.675
0.7	9.605	10.787	12.009	13.075	14.558
0.8	14.772	16.644	19.213	23.622	27.507
0.9	24.192	26.087	28.669	35.847	42.288
1	20.584	24.069	29.311	34.786	37.906

* x_2 is mass fraction of THP in TWM in the absence of ABN401
* Standard uncertainties , $u(T)$ = 0.05 K

Table S5. Activity coefficients (γ_1) of drug in various TWM mixture at 298.15 to 318.15 K.

w_2	γ_1				
	T = 298.15 K	T = 303.15 K	T = 308.15 K	T = 313.15 K	T = 318.15 K
0	887.91	609.19	431.99	314.50	211.50
0.1	237.99	174.55	124.77	94.99	68.33
0.2	156.04	102.64	68.81	49.18	39.19
0.3	90.04	52.07	35.62	24.62	16.71
0.4	31.51	25.12	17.64	10.31	5.98
0.5	12.91	7.43	5.00	3.35	2.61
0.6	5.35	3.80	2.70	1.95	1.50
0.7	2.61	1.89	1.39	1.06	0.79
0.8	1.70	1.23	0.87	0.59	0.42
0.9	1.04	0.78	0.58	0.39	0.27
1	1.22	0.85	0.57	0.40	0.30

* w_2 is mole fraction of THP in TWM in the absence of ABN401

Table S6. Thermodynamic parameters of drug dissolution in solvents at the harmonic temperature of 308.15 K.

Solvents	ΔH° KJ mol^{-1}	ΔG° KJ mol^{-1}	ΔS° J.mol^{-1}.K^{-1}	R^2
Water	24.92	31.89	–22.64	0.9981
Methanol	38.63	24.65	45.39	0.9986
Ethanol	49.24	19.94	95.14	0.9992
1-Propanol	18.54	17.91	2.04	0.9974
2-Propanol	37.51	19.91	57.16	0.9982
1-Butanol	12.45	17.76	–17.22	0.9993
2-Butanol	41.48	18.51	74.56	0.9961
Acetonitrile	11.78	18.59	–22.10	0.9967
Acetone	15.80	17.28	–4.81	0.9959
Ethyl acetate	8.99	18.54	–31.01	0.9983
THP	25.03	14.99	32.60	0.9987

* Standard uncertainties , $u(\Delta H^{\circ})$ = 0.14, $u(\Delta G^{\circ})$ = 0.23, $u(\Delta S^{\circ})$ = 0.26

Table S7. Apparent thermodynamic parameters for dissolution behaviour of drug in TWM mixture.

w_2	ΔH° KJ mol^{-1}	ΔG° KJ mol^{-1}	ΔS° J.mol^{-1}.K^{-1}	R^2
0	41.94	31.90	32.59	0.9976
0.1	40.48	28.70	38.24	0.9971
0.2	38.30	27.26	35.84	0.9978
0.3	29.62	25.47	13.47	0.9975
0.4	29.00	23.22	18.76	0.9981
0.5	26.61	20.51	19.80	0.9970
0.6	25.03	18.88	19.96	0.9969
0.7	24.92	17.19	25.09	0.9968
0.8	23.58	15.84	25.12	0.9976
0.9	21.52	14.73	22.04	0.9981
1	19.07	14.99	13.24	0.9988

w_2 is mole fraction of THP in TWM in the absence of ABN401.
* Standard uncertainties , $u(\Delta H^\circ)$ = 0.15, $u(\Delta G^\circ)$ = 0.29, $u(\Delta S^\circ)$ = 0.09

Table S8. Stability results for ABN401 nanosuspension formulation.

Formulations		Concentration (%, w/v)	Particle size (z-average, nm)			Particle size (d_{90}, nm)			Zeta potential (mV)
			Initial	3 days	7 days	Initial	3 days	7 days	
F1	Kollidon® VA / Kollidon® K12 / Lutrol® F127	1.0/0.5/1.0	54.9	156.3	278.2	186.4	531.3	568.2	–35.26
F2	Kollidon® VA / Kollidon® K12 / HPβCD	1.0/0.5/1.0	43.0	84.9	105.7	183.1	420.8	476.2	–43.07
F3	Kollidon® VA / Kollidon® K12 / PEG 6000	1.0/0.5/1.0	53.1	255.2	396.1	168.3	505.3	531.6	–34.57
F4	Kollidon® VA / Kollidon® K12/ SLS	1.0/0.5/0.1	120.1	184.7	218.3	226.1	929.4	1086.0	–40.19

Table S9. Materials used in the experiments

Name	Molecular formulae	Molar mass (g ·mol^{-1})	Mass fraction purity (%)[a]	Analysis method	Source
ABN401	$C_{29}H_{34}N_{12}O$	566.66	99.9	HPLC[b]	Abion Inc.
Methanol	CH_4O	32.04	99.9	GC[c]	Avantor Performance Materials
Ethanol	C_2H_6O	46.07	99.9	GC	Avantor Performance Materials
1-Propanol	C_3H_8O	60.10	99.5	GC	Daejung Chemical & Metals Co., Ltd.
2-Propanol	C_3H_8O	60.10	99.7	GC	Daejung Chemical & Metals Co., Ltd.
1-Butanol	$C_4H_{10}O$	74.12	99.5	GC	Junsei Chemical Co., Ltd.
2-Butanol	$C_4H_{10}O$	74.12	99.5	GC	Daejung Chemical & Metals Co., Ltd.
Acetonitrile	C_2H_3N	41.05	99.9	GC	Avantor Performance Materials
Acetone	C_3H_6O	58.08	99.8	GC	Daejung Chemical & Metals Co., Ltd.
Ethyl acetate	$C_4H_8O_2$	46.07	99.5	GC	Junsei Chemical Co., Ltd.
THP	$C_6H_{14}O_3$	134.17	99.4	GC	Gattefosse Co.,

[a] Mass fraction purity values were provided by the suppliers.
[b] High performance liquid chromatography
[c] Gas chromatography

Table S10. Particle size of nanosuspension prepared using different drug concentrations in THP and various ratios of solvent/anti-solvent using Kollidon® VA64/ Kollidon® K12/ HPβCD.

Mole fraction in THP	Ratio of solvent/ Anti-solvent	Mole fraction in TWM mixture	Particle size (z-average, nm)	Particle size (d_{90}, nm)
0.04	1/4	0.2	68.6	174.9
0.04	1/9	0.1	43.0	183.1
0.08	1/4	0.2	70.0	194.1
0.08	1/9	0.1	57.5	192.2

Table S11. Particle size of nanosuspension prepared using various polymers and stabilizer combination.

	Formulations	Concentration (%, w/v)	Particle size (z-average, nm)	Particle size (d_{90}, nm)
F1	Kollidon® VA/ Kollidon® K12/ Lutrol® F127	1.0/0.5/1.0	54.9	186.4
F2	Kollidon® VA/ Kollidon® K12/ HPβCD	1.0/0.5/1.0	43.0	183.1
F3	Kollidon® VA/ Kollidon® K12/ PEG 6000	1.0/0.5/1.0	53.1	168.3
F4	Kollidon® VA/ Kollidon® K12/ SLS	1.0/0.5/0.1	120.1	226.1

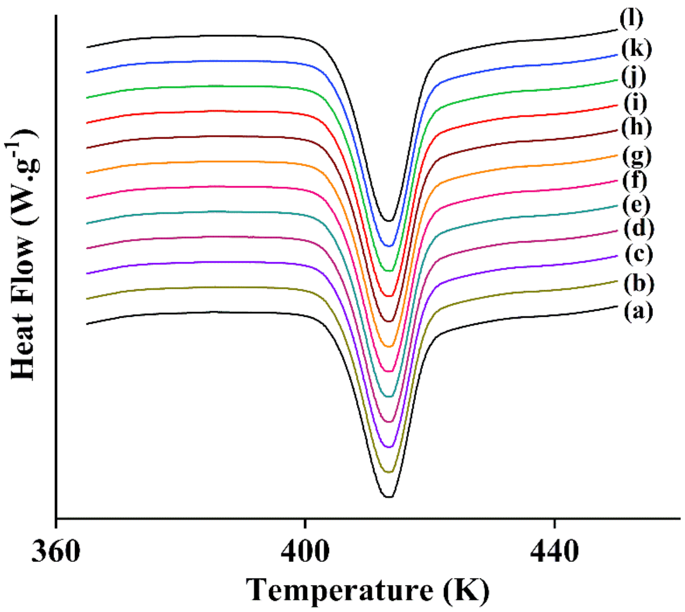

Figure S1. DSC thermograms of drug alone (a), drug - recovered from water (b), methanol (c), ethanol (d), 1-propanol (e), 2-propanol (f), 1-butanol (g), 2-butanol (h), acetonitrile (i), acetone (j), ethyl acetate (k), and THP (l).

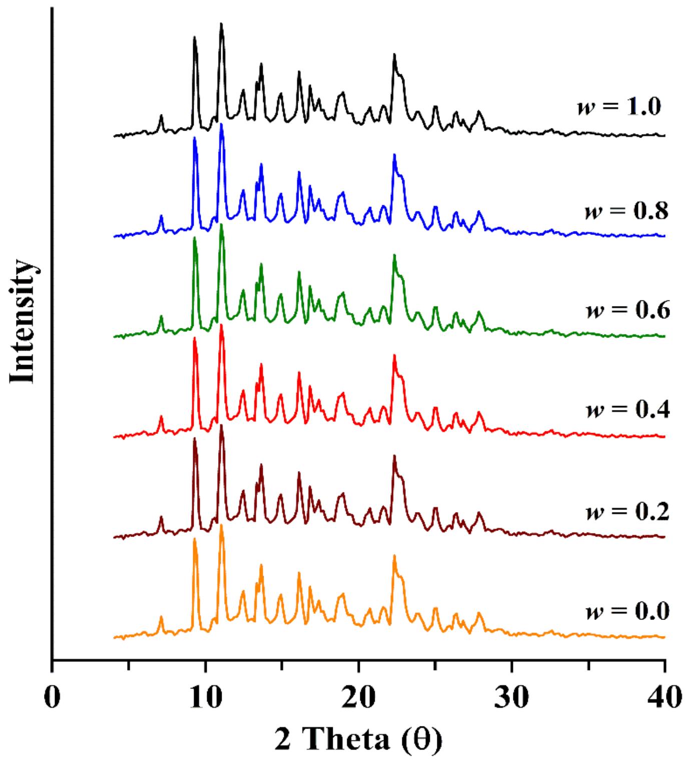

Figure S2. PXRD patterns of drug before and after solubility experiments in THP (*w*) + water (1-*w*) mixed solvents.

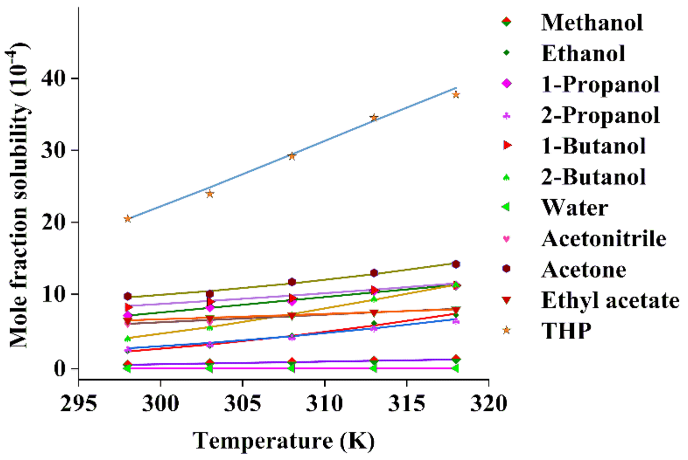

Figure S3. Experimental and calculated mole fraction solubility of ABN401 on various organic solvents based on modified AM equation. Solid lines denote the calculated solubility.

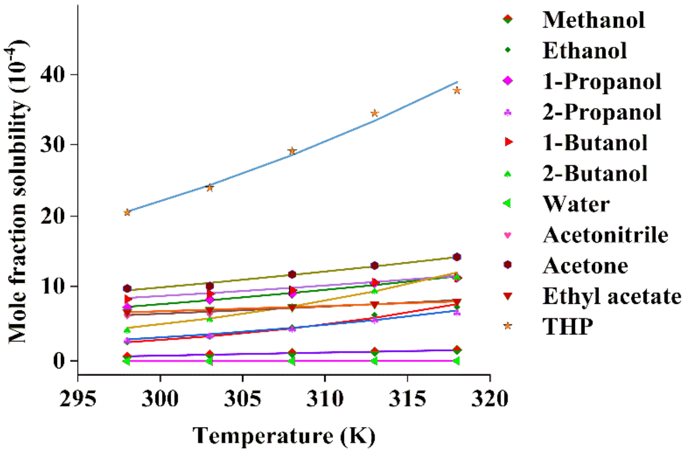

Figure S4. Experimental and calculated mole fraction solubility of ABN401 on various organic solvents based on ideal model. Solid lines denote the calculated solubility.

Reference

S1. Lee, S.-K.; Sim, W.-Y.; Ha, E.-S.; Park, H.; Kim, J.-S.; Jeong, J.-S.; Kim, M.-S. Solubility of bisacodyl in fourteen mono solvents and N-methyl-2-pyrrolidone+ water mixed solvents at different temperatures, and its application for nanosuspension formation using liquid antisolvent precipitation. *J. Mol. Liq.* **2020**, 113264. https://doi.org/10.1016/j.molliq.2020.113264

S2. Jessop, P. G.; Jessop, D. A.; Fu, D.; Phan, L. J. G. C. Solvatochromic parameters for solvents of interest in green chemistry. **2012,** 14, 1245-1259.

S3. Marcus, Y. The properties of organic liquids that are relevant to their use as solvating solvents. *Chem. Soc. Rev.* **1993,** 22, 409-416.
